# A sample of non-nutritive sucking habits (pacifier and digit) in portuguese children and its relation with the molar classes of angle

**DOI:** 10.4317/jced.55284

**Published:** 2018-12-01

**Authors:** Sónia-Cristina-Silva Machado, Maria-Cristina Manzanares-Céspedes, Joaquim Ferreira-Moreira, José-Júlio Ferreira-Pacheco, Paulo-Alexandre-Martins-Abreu Rompante, Josep-Maria Ustrell-Torrent

**Affiliations:** 1 Graduation. DDS, Doctoral student at the University of Barcelona. Advanced Institute of Health Sciences University (IUCS), Portugal; 2 MD, PhD. Professor Titular de Universidad, Faculty of Medicine and Sciences of Health. University of Barcelona; 3 MD, PhD. Associate Professor. Administrative positions: Director of the Dentistry School and the Director of Dentistry. Department of the IUCS (University Institute Health Sciences), Portugal; 4 President of the Pedagogical Council IUCS. Head of the Medical. Service and Oral Surgery. Department of Dental Sciences. Clinical Director of the Clinical Unit of CESPU (IUCS); 5 MD, PhD. Paediatric Dentistry, Advanced the University Institute of Health Sciences (IUCS). Portugal; 6 MD, PhD. Associate Professor. Vice Dean School of Dentistry. Oral Health and Masticatory System Group (Bellvitge Medical Research Institute) IDIBELL. L’Hospitalet (Barcelona) Spain

## Abstract

**Background:**

Little is known about the effect of non-nutritive sucking habits (pacifier and digital sucking) in the prevalence of molar Class in mixed dentition. The aim of this study was determinate the relation between non-nutritive sucking habits, and Angle´s molar Class, in the horizontal plane, and it´s relation with gender. A convenience sample of 326 children with ages between 6 and 12 years was selected from three schools of Oporto.

**Material and Methods:**

To collect the epidemiologic data, was used a method recommended by the WHO. An indirect questionnaire about the medical history, dental habits, was used. It was adapted from Sanchez-Molins and validated by Clinical Dental III of Integrated Dental University Institute Health Sciences, Gandra, Portugal.

**Results:**

In this study, 326 infants were examined in order to determine the prevalence of non-nutritive sucking habits. Only 45 observed children did not mentioned any kind of non-nutritive sucking habit; the remaining 281 children mentioned at least one potential bad habit. Children with non-nutritive sucking habits show a higher molar Class II percentage in females, while molar Class III is more frequent among males compared with children with no sucking habits.

**Conclusions:**

Children with non-nutritive sucking habits, presented a higher-Class II prevalence with statistically significance. It was detected a direct relationship between Angle´s molar Class and gender.

** Key words:**Finger sucking, pacifier sucking, Angle Class malocclusion.

## Introduction

Non-nutritive sucking habits are among the etiological local factors that many cause malocclusions.

The commonest form of non-nutritive sucking (NNS) is digit sucking. Several studies suggested that fatigue, boredom, excitement, hunger, fear, physical and emotional stress, and insufficient satisfaction of sucking need in infancy, are situations that could stimulate digit sucking habits (1).

The studies of relationships of non-nutritive sucking habits (digit sucking and/or pacifier) and Angle Class molar in the mixed dentition are less common.

Among studies conducted of the mixed dentition, a transversal study of sample of 326 Portuguese children from 6 to 12 years old. These, 281 cited one or both habits.

Children’s habit of sucking on a pacifier and/or digit (finger) is universal, but there are differences between countries and cultures. With the maintenance of this habit there are consequences both on the inside and outside the orofacial area (2). The sucking habit on a pacifier usually disappears spontaneously around the third year, as the child grows older and interest in other activities is developed (3). Nonetheless, the habit of sucking on a digit prevails, often until 7 or 8 years old. Breaking this habit tends to be harder if the sucked object is part of the child’s body and the habit might also be connected to more serious emotional and psychological matters, especially in older children (3).

Tooth eruption is a continuous biological process by which developing teeth emerge through the jaws and the overlying mucosa to enter into the oral cavity. Tooth eruption time and sequence are important factors in dental treatment planning, particularly in orthodontics (4).

The activity of non-nutritive sucking should be diagnosed in a timely manner in order to reduce the development of posterior cross bite, anterior open bite, and Class II molar relationship (5).

The purpose of the present study was to assess the relationship between non-nutritive sucking habits and different Angle´s Class molar, using the transversal study in a convenience sample.

## Material and Methods

To collect the epidemiologic data, according to our aims, was used the method recommended by the World’s Health Organization (WHO) (6).

The method used to evaluate the children’s tooth eruption level was the one described by Carr (1962) (7) and Barbería (8) which considers a tooth as erupted when it emerges through the gum and becomes visible in the oral cavity. This is a simple, non-invasive criteria that doesn’t require complementary exams and facilitates the calibration of the survey.

Thus, we have objective data collected by the author, and subjective data provided by parents.

The children’s examination and observation as well as the data collected was made by a single observer. The observer used the intra examiner calibration methodology according to the epidemiological surveys manuals recommended by the methodology (6). A “kappa test (k)” was applied to determine the variations intra observer, since the observer performed two measures of the same variable in the same child.

For data collection, the participants were examined sitting, under natural light and used disposable mouth mirror. All measures were taken with a calibrated millimeter standard ruler.

Clinical observation of patients and respective data collection were carried out by me, single observer.

49 children randomly selected, which correspond to 15% of children in the study population to assess the error of the examiner (6). “T test” was used for paired data, since the observer performed two measurements of the same variable in the same child.

The following observation was made in accordance with a record whose information included; type of teeth, number of cavities, evaluation of vertical intermaxillary relationship parameters, horizontal and cross extracted deciduous teeth or exfoliated and permanent teeth erupted by quadrant.

Subsequently the registration was made in accordance with a form designed especially for the adapted effect of the WHO data set, validated in the Course of Clinical pediatric dental III, the Master of Dental Medicine of the University Institute Health Sciencies, Gandra,Portugal.

To confirm the data obtained, we carried out a survey to parents or guardians to obtain information about medical-dental and habits, syndromes, congenital diseases, childhood diseases, trauma, surgery, device pathology respiratory and oral habits, pacifier use and thumb sucking.

Confidentiality was guaranteed by assigning numeric codes to each of the participants as well as any other information that could indicate the identity of the participants. Later, the data were stored on personal computer security lock after being transfered to a Microsoft Office Excel ™ tab sheet.

Similarly, the patient’s right to be informed of the conditions of research has been maintained, and the individual results provided to all interested participants.

The convenience sample was obtained in the institutions of the metropolitan area of Oporto. The selection criteria included children of both male and female genders, aged 6 to 12, Portuguese, caucasians and institutional affiliation at least one parent or guardian.

Thus, 326 individuals of male and female gender, regardless of their socioeconomic status were included for evaluation and statistical analysis

The study sample (n = 326) consists of children from 6 to 12 years of age, of which 180 (55%) were female and 146 (45%) are male gender. In this study 86% of children have at least one non-nutritive sucking habit ( Table 1- Table 3).

**Table 1:**
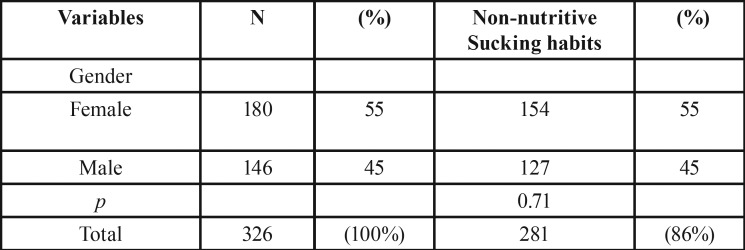
Characteristics of the sample (n=326).

**Table 2:**
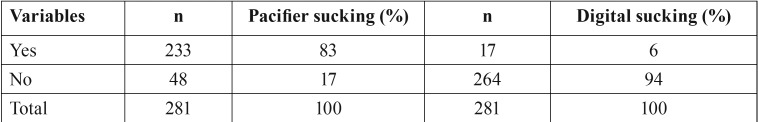
Non-Nutritive sucking habits (n= 281).

**Table 3:**
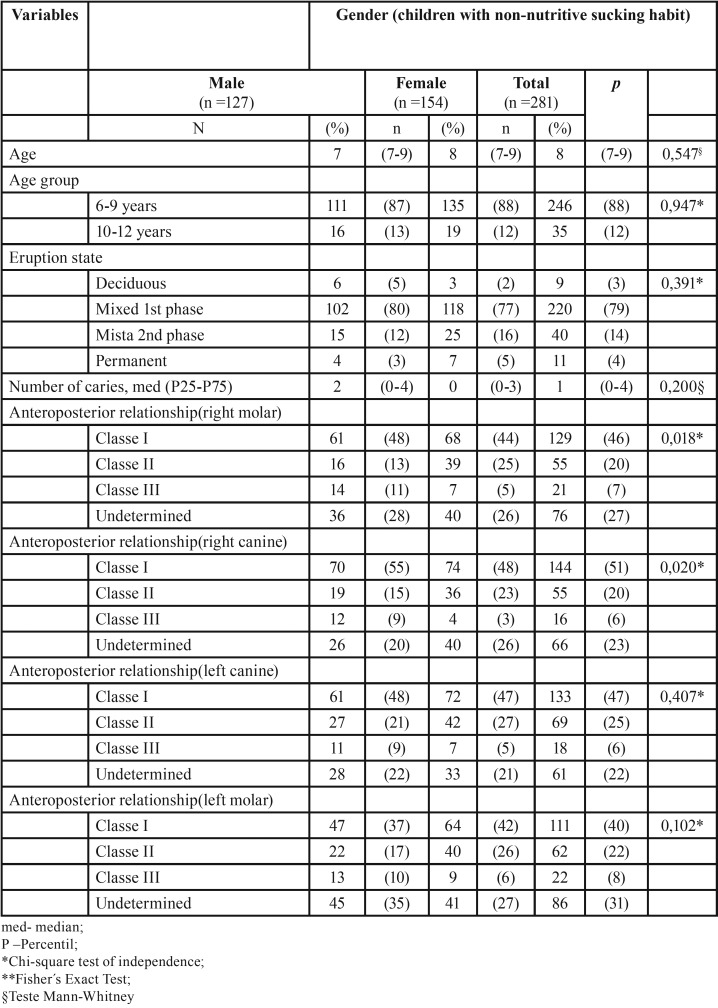
Comparison of socio-demographic data and ontological sample data in a study with non-nutritive sucking habits (n = 281) according to gender.

The statistical analysis was made using the SPSS program version 17a. We used the most appropriate descriptive statistics to the study: medias, medians, frequency, distribution, variation and corresponding pattern’s deviation calculations.

The frequency of non-nutritive sucking habits and appearance of changes in the chronology of eruption were analyzed in relation to the total number of subjects and by gender.

The categorical variables were described by absolute frequencies (n) and relative frequencies (%), while the continuous quantitative variables were described using the medians, 25th percentile and 75th percentile, since the distribution of these is asymmetric.

The Chi-square independence test, was used to investigate the association between categorical variables.

In the beginning of the study was requested and granted permission by each of the directions of the institutions where the research took place.

Parents or guardians that agreed to voluntarily participate in the investigation were subjected to the process of informed consent. To them was handed a document containing in writing information about the objectives of the study and the research protocol which had to be returned signed.

The refusal to voluntarily participate in the study received the proper dental care without any form of discrimination.

The author saw no need to ask permission to the respective Committees of Ethics, in so far as it does not violate the integrity and privacy of the participants. Moreover, the author states that no conflicting interests in carrying out this investigation.

So it does not produce any violation of ethical principles in relation to medical investigation that includes human subjects, as stated in the Declaration of Helsinki adopted by the World Medical Association in 2004 .

## Results

The group of children with nonnutritive sucking habits (pacifier and digit) have a relationship between gender and right Angle molar Class (*p*= 0,018) ( Table 4).

**Table 4:**
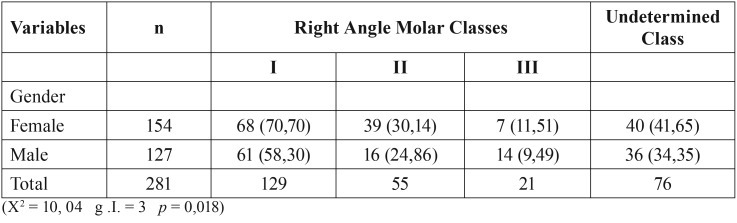
Right Angle Molar Class and non-nutritive sucking habits (n =281).

They are significant in this relationship “Class II” and “Class III”. Increased female Class II and Class III increased in males ( Table 4).

If we take the group of indeterminate children, the results stay the same, no variations (*p*= 0,019).

If we remove the indeterminate class, the results remain the same unvaried ( Table 5).

**Table 5:**
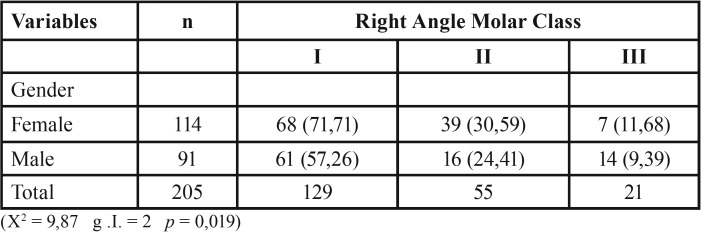
Right Angle Molar Classes and non-nutritive sucking habits without Undetermined Class (n =281).

## Discussion

The study sample (n = 326) consists of children from 6 to 12 years of age.

The age group of 6 to 12 years was chosen due to the maturation of children, allowing for greater cooperation with regard to the acceptance of the physical examination.

Our study showed than in children with non-nutritive sucking habits (86%) ( Table 1), have an anteroposterior relationship of Class II right molar the larger in females, while there is a higher percentage of Class III right molar in males (*p*=0,018, *p*=0,019) ( Table 4, Table 5).

That is in agreement with a recent study (Franco-Varas and Gorritxo, 2014) (9), where a significant increase of malocclusion in the primary dentition of children who prolonged the pacifier-sucking habit was referred. Moreover, it was described that, if this habit was abandoned early, anterior open bites improved, while posterior crossbite clutch remained or even got worse. In a study on nipo-brazilian children aged between 2 and 6 years old with mixed dentition, where the connection between the non-nutritive sucking habits-pacifier and/or digit and anteroposterior occlusal relationships was evaluated, a higher percentage of children with molar Angle Class I, then with Class II and fewer with Class III was described (Montiel-Jaime, 2004) (10).

Comparing the obtained results with results from international literature, specifically with the ones from Iberian peninsular populations (Hernandez *et al.*, 2002) (11), has made possible to determine the inter-dental relationship between gender and the Angle Class in the population with non-nutritive sucking habits. We observed that the anterior posterior molars (25% on the right, 26% on left on females; 13% on the right, 17% on the left on males) identical to Angle Class II, are higher on females than on males. In situations identical to molar Angle Class III (5% on the right, 6% on the left on females; 11% on the right, 10% on the left on males) the changes are higher in males than in females.

Malocclusion in the mixed dentition was associated with prolonged nonnutritive sucking habits (12).

There was no significant association between breastfeeding or bottlefeeding duration and malocclusion in the deciduous dentition, but the exclusive breastfeeding reduce the non-nutritive sucking habits (13).

Consequently, we can suggest a direct relationship between Angle Classes II and III and gender, since in our population Classes II are more frequent in females and Classes III in males.

Some limitations were observed in this study, in this case we used of a random sample, it is assumed that 

the representative lacks criteria inherent in a population sample.

The methodology used of a random sample is legitimate. It´s possible the obtained from the data analysis elations, can be clarifying the doubts that justify the preparation of this study. It´s recognized that their extrapolation to the general population lack the necessary precautions.

The results of this investigation, obtained by a simple study, transversal and noninvasive, provide information to any health professional for better pediatric knowledge concerning external factors that interfere with getting a harmonious and healthy oral cavity.

The persistence of habits and dysfunctions during the first years of life are factors that aggravate the malocclusion.

This study in addition to being a original theme, can contribute to help the professional to the most appropriate treatment for each case.

## Conclusions

Based on this study’s results, it can be concluded that the non-nutritive sucking habits, digit and pacifier, is very high (86%).

It was also detected a direct relationship between Angle molar Classes II and III and gender.

Classes II molar are more frequent in females and Classes III molar in males.
